# Editorial

**Published:** 1874-01

**Authors:** 


### Editorial.

### *Rush Medical College—Spring Course.*

In the present No. of the JOURNAL will be found the Fifteenth Annual Announcement of the Spring Course of Lectures of Rush Medical College.

It will be remembered that one year ago the Faculty decided to reorganize this department of the College, and to that end, threw open the chairs to public competition. The selections made from this concours, appear to have justified the confidence reposed in them, and the experiment (novel in this country) of filling such positions upon the basis of merit alone, has demonstrated its wisdom by its success.

The summer class of 1873 was large, its average of capacity and diligence exceptionably high, and its members are conspicuous in the present winter class. The prospects are that the summer class of 1874 will exceed in numbers that of 1873 very largely, and the advantages held out to them will be correspondingly increased.

The second concours held at the College during the past week resulted in the selection of the successful competitor, Dr. Albert B. Strong, to fill the vacant lectureship of Materia Medica and General Therapeutics. H.

### *Bogus Diploma Traffic.*

The *New York Medical Record*, under date of Dec. 1, has the following:

"The bogus diploma institution in Philadelphia is likely to have a chance to vindicate what the faculty please to call their "fair fame." Mayor Stokely, of that city, has been informed that the State Supreme Court has issued a writ of *quo warranto* against the said institution, returnable at the next session of the court in that city. Of course, the President, Dean, and others of the "University," are anxious for an investigation, but we imagine that they are not quite so clamorous for the justice that may follow. The law, however, gives the greatest swindlers a chance for escape."

We have just received a number of the organ of this "University," and have never seen a more impudent summary of quackery and general rascality in print.

It is stated elsewhere, that the graduates of this concern are to be found in "Chicago" and other places designated. We hope that a list of these "graduates" with their residence may be published, not only in the medical journals but in the secular press likewise. A little gratuitous advertising of these vagabonds will subserve the public interests materially. This is the second time that the JOURNAL has taken occasion to direct attention to this den of thieves, in view of which fact, the sending of their pamphlet is decidedly cool.

Will not our Philadelphia exchanges favor us with a list of the alumni (!) of this "University" who have honored (?) this city with their presence? H.

### *"Crowner's 'Quest."*

Since our last issue we have had a new edition of the old story, "One more unfortunate . . . . gone to her death," or rather done to death with that most diabolical weapon, the abortionists' bougie.

It is perhaps only just to the jury of inquest to believe that they acted in good faith in presenting Dr. Earle and the husband, Hill, to the grand jury as principal and accessory to the murder of the woman Hill, but a little consideration would have shown them the folly of such a course, and the impossibility of securing an indictment of the accused parties upon such a charge. The legal definition of murder, being, we believe, the felonious destruction of human life with malice aforethought, there was not the slightest evidence adduced at the inquest to show that either of the accused parties, or indeed that any one else, ever designed the killing of Mrs. Hill; on the contrary, the presumption is strong that this was an act of the drama not set down in the programme, and would have been prevented had it been possible. That the woman died from the results of an abortion was clearly established, and that the perpetrators of the crime might readily have been detected, and should have been punished to the full extent permitted by the technicalities of the law, is also certain; but we cannot evade the conviction that the attention of the jury was occupied with the incidental features of the horrible transaction, to the exclusion of its essential element.

The complaint is a standing one, that abortionists cannot be convicted; the reason is obvious, they are rarely arraigned, and still more rarely tried upon the true issue. If some poor woman is slaughtered by their lack of skill, they may perhaps, as in this case, be arraigned for her murder, and as in this case, cannot be convicted for lack of evidence to sustain the charge.

But we assert that there was *murder*, "*murder most foul*," murder sufficiently clear to satisfy the most minutely technical definition of the law, *i. e.*, the felonious killing of a human being with malicious intent. Until the statute law ceases to exclude the fœtus in utero from the category of human beings, until it throws its protection around them also, the massacre of the innocents will go on unchecked, and the pariahs of the profession will thrive and grow fat, and will pamper their declining years with their ill-gotten gains, will shelter their gray heads beneath pretentious roofs, loll their bloated carcasses in comfortable carriages, clothe their wives, their children and *even grand-children* in luxurious garments, all purchased with the price of blood, tribute to their surgical (!) skill, whose sole armamentum chirurgicum is the bougie.

Let physicians exempt from the rule of professional confidence all applications to produce abortions, by whomsoever made, and the public will soon be able to identify these excrescences upon the profession, already well known to the profession itself. H.

